# Evidence for systems-level molecular mechanisms of tumorigenesis

**DOI:** 10.1186/1471-2164-8-185

**Published:** 2007-06-20

**Authors:** Pilar Hernández, Jaime Huerta-Cepas, David Montaner, Fátima Al-Shahrour, Joan Valls, Laia Gómez, Gabriel Capellá, Joaquín Dopazo, Miguel Angel Pujana

**Affiliations:** 1 Bioinformatics and Biostatistics Unit, and Translational Research Laboratory, Catalan Institute of Oncology, IDIBELL, L'Hospitalet, Barcelona 08907, Spain; 2 Functional Genomics Unit, Bioinformatics Department, CIPF, Valencia 46013, Spain

## Abstract

**Background:**

Cancer arises from the consecutive acquisition of genetic alterations. Increasing evidence suggests that as a consequence of these alterations, molecular interactions are reprogrammed in the context of highly connected and regulated cellular networks. Coordinated reprogramming would allow the cell to acquire the capabilities for malignant growth.

**Results:**

Here, we determine the coordinated function of cancer gene products (i.e., proteins encoded by differentially expressed genes in tumors relative to healthy tissue counterparts, hereafter referred to as "CGPs") defined as their topological properties and organization in the interactome network. We show that CGPs are central to information exchange and propagation and that they are specifically organized to promote tumorigenesis. Centrality is identified by both local (degree) and global (betweenness and closeness) measures, and systematically appears in down-regulated CGPs. Up-regulated CGPs do not consistently exhibit centrality, but both types of cancer products determine the overall integrity of the network structure. In addition to centrality, down-regulated CGPs show topological association that correlates with common biological processes and pathways involved in tumorigenesis.

**Conclusion:**

Given the current limited coverage of the human interactome, this study proposes that tumorigenesis takes place in a specific and organized way at the molecular systems-level and suggests a model that comprises the precise down-regulation of groups of topologically-associated proteins involved in particular functions, orchestrated with the up-regulation of specific proteins.

## Background

In recent years, functional genomic and proteomic approaches have generated a vast quantity of data through which cellular processes, pathways and pathologies can be deciphered. In particular, microarray-based studies have provided genome-wide expression data for almost every type of human cancer [1]. As a consequence of genetic and molecular analyses, the sequence of events that contributes to certain types of human cancer, for example colorectal cancer [2], is relatively well characterized.

Although our understanding of the genetic determinants of tumorigenesis has been greatly enhanced by these approaches [3], other levels of molecular complexity have emerged [4-6]. Cancer arises from the consecutive acquisition of genetic alterations that, in general, can be recognized as the combination of the loss of function or transcriptional down-regulation of particular genes (tumor suppressor genes) and the activation or transcriptional up-regulation of other genes (oncogenes) [3]. Downstream of the genetic alterations are expression changes in many genes in cancer cells, mediated in part by the activation or inactivation of transcription factors [7,8]. It is thought, then, that genetic and molecular alterations promote tumorigenesis in the context of highly connected and regulated gene and protein networks [4-6,9]. Cellular transformation therefore requires dynamic interconnectedness, where specific changes in the information circuitry primarily dictated by up- or down-regulated genes activate or deactivate pathways and, finally, change the cell phenotype. In order to develop a systems-level understanding of cellular transformation it would therefore be necessary to determine the properties and organization of CGPs (proteins encoded by differentially expressed genes in tumors relative to healthy tissue counterparts) in cellular networks.

This study examines the topological properties of CGPs in the human interactome network. Wachi et al. [10] previously reported increased connectivity of differentially expressed proteins in lung cancer tissues, and Jonsson and Bates (2006) [11] reported differences in the global topological features of mutated cancer proteins relative to non-mutated proteins. However, there is no comprehensive study of different cancer types that examines both the local and global topological properties of CGPs and their organization relative to the structural integrity of the network and to molecular mechanisms of tumorigenesis. The results of these analyses suggest that CGPs are central to information exchange and propagation, and that their topological organization supports fundamental biological processes of neoplasia.

## Results

### Integration of interactome and cancer transcriptomes

**Figure 1:**
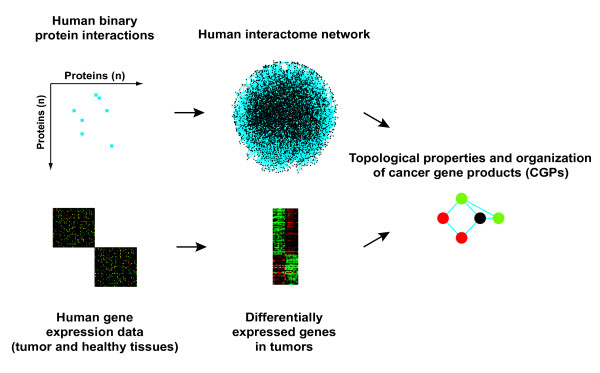
Study strategy. Integration of binary protein-protein interactions and gene expression data sets for the investigation of the topological properties and organization of cancer gene products (CGPs) in the human interactome network.

To investigate the systems-level organization of CGPs, we integrated interactome and cancer transcriptome data sets (Figure 1). The interactome data set contains compiled and filtered binary human protein-protein interactions from all currently available databases (HPRD, BIND, DIP, MINT, INTACT and MIPS; detailed in Gandhi et al. [12]). This data set is mainly derived from one-at-a-time experimentally demonstrated interactions compiled through a literature curation process [13], which suggests a high degree of reliability. The corresponding scale-free interactome network contains 7,388 proteins and 24,109 interactions, which follow a power-law distribution with an average degree of 6.52 (Additional file 1). The longest distance between any two proteins is 15 and the average distance is 4.50. This interactome network constitutes a scaffold in which different types of functional genomics data can be integrated to ascertain the coordinated function of proteins under particular conditions.

To analyze cancer transcriptomes, we chose data sets of high-incidence cancer types containing a large number of tumors and healthy tissue samples in order to obtain more consistent gene lists (data sets with at least 10 samples of each type). Four expression data sets were analyzed, corresponding to prostate, lung and colorectal samples [14-17] (Additional file 2). We first focused our analysis on the prostate data sets because they were independently generated and contain publicly available raw data, which meant an identical statistical methodology could be applied for differential gene expression detection and to replicate findings [14,15]. These data sets contain data for 50 healthy tissue samples each and for 52 and 38 tumor samples, respectively. Differentially expressed genes between healthy and tumor samples were then identified using an empirical Bayes moderated t-test and adjusting *P *values with a false discovery rate of 5%. Thus, 1,429 and 981 CGPs encoded by up- and down-regulated genes in prostate tumors were mapped in the interactome network, respectively. Up- and down-regulated gene sets overlapped between studies by 50.33% and 41.05%, respectively. Accordingly, both studies also showed a similar distribution of Gene Ontology (GO) [18] terms annotation in the complete gene ranking (Additional file 3), which essentially supports a good agreement between the expression data sets. The numbers of differentially expressed genes obtained in this analysis are consistent with the numbers given in the original publications. A comparison of healthy and tumor tissues is likely to reveal more dramatic expression differences than a comparison of tumor subtypes, thus identifying differentially expressed genes that are involved in all stages of the neoplastic process.

In order to extend the analysis to different types of CGPs, we used expression data sets derived from the study of lung samples (230 tumors and 17 healthy), which included different cellular types, and colorectal samples (18 tumors and 36 healthy) [16,17]. The lung expression data set was analyzed using the same statistical methodology as described for the prostate, while genes differentially expressed in colorectal tumors identified on a different microarray platform were taken from a public repository [19]. Sets of differentially expressed probes for each cancer type are detailed in (Additional file 2). Integration of the human interactome and cancer transcriptomes was then completed by matching GeneIDs.

### Centrality of CGPs

Analysis of the topological properties of CGPs in the interactome network was focused on centrality by measuring: i/ degree, which accounts for the total number of first interactions; ii/ betweenness, which accounts for the frequency with which a node in a network is found in the shortest path between any two other nodes; and iii/ closeness, which accounts for the proximity of a node to all other nodes in a network. To determine the significance of each measure, we compared the median of CGPs to the median of the total of nodes in the network using the Mann-Whitney *U *test. We also compared the results to equivalent randomly selected protein sets in the interactome.

**Figure 2:**
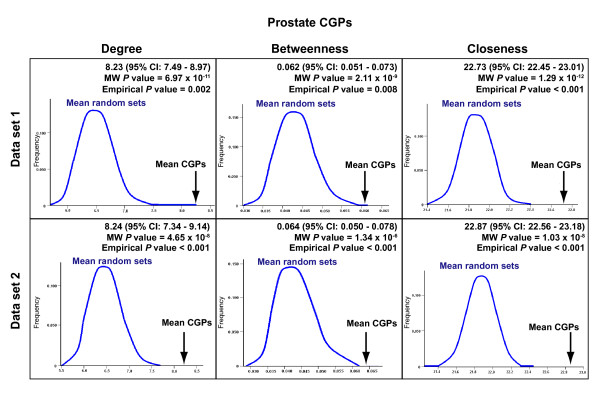
Centrality of CGPs. Results of the Mann-Whitney *U *test (MW) are shown at the top right in each box. Results of comparing each centrality measure between prostate CGPs (vertical arrow; mean value) and 1,000 equivalent randomly selected protein sets (curves; mean values) (data sets 1 [14] and 2 [15]) are also shown. CGPs mean values and 95% confidence intervals (CI), as well consequent empirical *P *values are shown.

The analysis of prostate CGPs revealed higher values for degree, betweenness and closeness than in the complete interactome set (Mann-Whitney *U *test *P *values < 10^-5^) or equivalent randomly selected sets (empirical *P *values < 0.01) (Figure 2 and Additional file 4). The results for the two prostate expression data sets were concordant. Importantly, higher values of centrality for lung and colorectal CGPs were also observed (Additional file 4). These results indicate that centrality in the interactome network is a common property of proteins encoded by differentially expressed genes in tumors relative to healthy tissue counterparts.

To further examine the topological properties of CGPs, we analyzed the manner in which they are related to their neighbors by examining their constraint, which accounts for the dependency of a node on its neighborhood. CGPs showed significantly lower average values of this measure (Mann-Whitney *U *test *P *values < 10^-7^; empirical *P *values < 0.01) (Additional file 4). CGPs therefore appear to act independently of their neighborhood, which supports the importance of these products in terms of information exchange and propagation within the interactome network studied.

Following this, we analyzed whether the topological properties of CGPs were mainly determined by one specific type of differentially expressed gene (i.e. up-regulated or down-regulated). This analysis highlighted that centrality is a property consistently found in down-regulated CGPs, while results for up-regulated CGPs were not conclusive (Additional file 5). The fact that up-regulated CGPs do not show consistent centrality measures could be the result of technical or biological differences between studies, for example that prostate tumor samples were collected at different stages [14,15]. On the other hand, this observation might also suggest that down-regulation plays a major role in tumorigenesis at the interactome network level.

Possible centrality differences between cancer subtypes were investigated by analyzing the lung data set according to the pathological description of tumors (adenoid, carcinoid, and squamous) [17]. Overlaps of 50.55%, 74.94% and 50.48%, respectively, were observed for adenoid-carcinoid, adenoid-squamous, and carcinoid-squamous down-regulated CGPs sets. In this case, all three subtypes showed centrality measures consistent with the analysis of prostate and colorectal down-regulated CGPs (Additional file 4). Once again, up-regulated CGPs showed heterogeneity of average values and value distributions.

### Centrality analysis using different sets of experimentally- or computationally-generated interactions

Comparison of publicly available protein-protein interaction repositories has revealed small, although significant, overlaps and considerable selection and detection bias [20,21]. To evaluate the consistency of the above results, we performed similar centrality analyses using three different sets of experimentally- or computationally-generated interactions: i/ *in vivo *experimental interactions only; ii/ interactions with two or more experimental evidences as compiled by Gandhi et al. [12] (interactions found *in vivo *and/or *in vitro*, including yeast two-hybrid interactions); and iii/ computationally-generated interactions using a homology-based method [22]. This final data set was carefully validated using true positive interactions sourced from the HPRD database and false positive interactions for proteins localized in incompatible cellular compartments based on Gene Ontology (GO) annotations [11]. Using these three interactions sets, the number of nodes and edges in each network were 6,022 and 15,990; 5,009 and 9,950; and 10,691 and 57,846, respectively.

Centrality was then examined in each network for CGPs of the two prostate cancer data sets, the lung cancer data set including three pathological sub-classes, and the colorectal cancer data set referred to above, distinguishing between down- and up-regulated CGPs. Importantly, the results of these analyses are fully consistent with increased local and global centrality and with lower constraint of CGPs, particularly for down-regulated CGPs (Additional file 6). In addition, the results using the homology-based network also showed increased centrality and lower constraint for up-regulated CGPs. This observation may be due to the higher number of nodes and edges in the network, which could diminish sampling errors relative to the anticipated complete interactome or, in contrast, to an unknown intrinsic bias of the homology-based method. Overall, analysis of the three interactome data sets further supports the hypothesis that high centrality is a fundamental property of CGPs.

### CGPs attack and interactome structure integrity

To better understand the relative importance of each centrality measure for CGPs, a strategy was used that consisted of determining the structural integrity of the interactome network after removing nodes with different topological characteristics [23-25]. We calculated the number of proteins remaining in the main component of the network (i.e., the part containing the largest number of connected proteins) after removing CGPs, selected nodes with the same degree distribution as CGPs but with lower values of betweenness and closeness centrality, and, in extreme cases, the hubs (proteins with the highest degrees).

**Figure 3:**
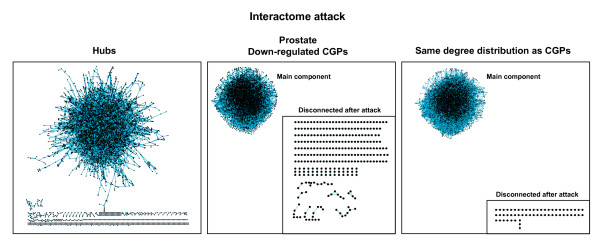
CGPs attack and interactome network structure integrity. Interactome network examples after removing an equivalent number of hubs, prostate down-regulated CGPs (data set 1 [14]) or selected proteins with the same degree distribution as CGPs, but with lower values of betweenness and closeness. Disconnected nodes from the main component are shown in inset to emphasize the difference between CGPs and selected proteins.

As expected from the association between centrality and vulnerability [23,24], removing CGPs had a lesser effect on structure integrity than did hubs removal. However, removing CGPs always produced a more dramatic effect than removing selected nodes with the same degree distribution but with lower values of betweenness and closeness. The number of proteins remaining in the main component was consistently smaller when CGPs were removed than when these selected nodes were removed (Figure 3 and Table 1). After deleting *n *nodes, the size of the main component is not only reduced by *n *but also by other nodes that are attached to CGPs. For example, removing 795 down-regulated prostate CGPs reduced the total number of nodes in the main component by 1,026 (7,092 to 6,066), while removing 795 proteins with the same degree distribution but with lower values of betweenness and closeness reduced the total number of nodes by 682 (7,092 to 6,410). Although the differences affect a small percentage of nodes in the main component (5.6% to 1.5%), the same tendency was observed for all the up- and down-regulated CGP sets examined (12 in total). These results suggest that the positions of both types of CGPs in the interactome network are more important than their degree distributions reflect.

**Table 1:** Interactome attack

			Number of nodes remaining in the main component after selective removal of:			
				
		N*	Hubs	CGPs	Same degree nodes	Difference**
Prostate cancer						
Data set 1	Down-regulated	795	4,092	6,066	6,410	344
	Up-regulated	634	4,616	6,294	6,610	316
Data set 2	Down-regulated	574	4,826	6,312	6,591	279
	Up-regulated	407	5,346	6,586	6,807	221

Lung cancer						
Adenoid	Down-regulated	476	5,112	6,460	6,719	259
	Up-regulated	187	6,134	6,830	6,966	136
Carcinoid	Down-regulated	786	4,119	5,965	6,368	403
	Up-regulated	518	5,002	6,421	6,736	315
Squamous	Down-regulated	458	5,171	6,479	6,716	237
	Up-regulated	525	4,974	6,380	6,640	260

Colorectal cancer						
	Down-regulated	164	6,220	6,849	6,960	111
	Up-regulated	289	5,726	6,709	6,858	149

*Number of CGPs mapped on the complete human interactome (i.e. number of nodes removed in this analysis)

**Main component difference between removing nodes with the same degree distribution as CGPs but with lower values of betweenness and closeness, and CGPs

### Topological and functional association of CGPs

Using the experimentally-based data sets, analysis of the level of inter-connection with neighboring proteins through the average clustering coefficient (CC) and by examining cliques (i.e. fully connected network sub-graphs) did not reveal significant over-representation of CGPs when corrected by multiple testing (not shown). In agreement with the lower constraint values observed, these observations suggest that CGPs perform their systems-level function principally by exploiting centrality (degree, betweenness and closeness), although not by virtue of being highly inter-connected in their neighborhood. However, the same analysis using the homology-based data set revealed significant differential CC values for both down- and up-regulated CGPs in different tumor types (Additional file 6). Larger, experimentally-based data sets are therefore needed to clarify the reasons for this discrepancy.

Next, we assessed whether the average network distances between CGPs were lower than the average in the main component. Thus, we determined the shortest distance between CGPs and compared this to the shortest distance between any two proteins in the main component. Lower distances were observed between CGPs – up-regulated, down-regulated, or both – when compared to the average distance in the main component (4.09 – 4.34 against 4.50, respectively) (Table 2). Accordingly, the maximum distances between CGPs were always found to be smaller than the maximum distance between any two proteins in the main component (10–12 against 15, respectively). These results suggest the topological association of CGPs regardless of CC or up/down-regulation.

**Table 2:** Topological association of prostate CGPs

			Network distance	
			
		N*	Shortest (average)	Maximum
Data set 1	Down-regulated	773	4.27	12
	Up-regulated	608	4.09	10
	All	1,381	4.22	12
Data set 2	Down-regulated	565	4.14	11
	Up-regulated	392	4.34	11
	All	957	4.23	12

Main component		7,092	4.50	15

*Number of CGPs in the main component

**Figure 4:**
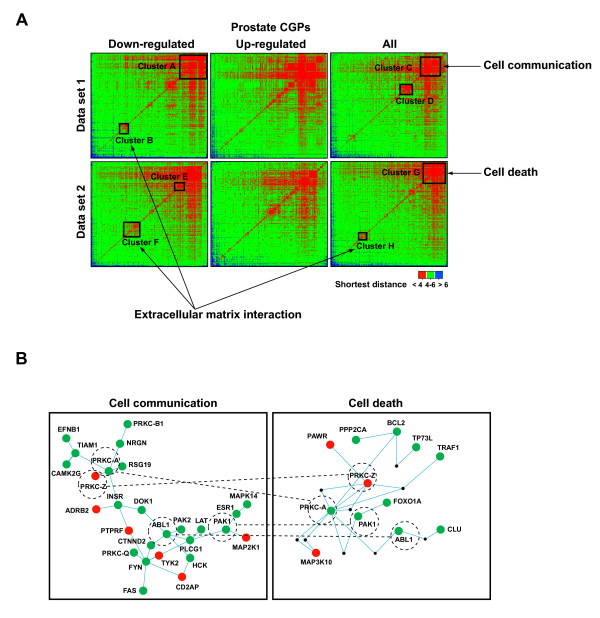
Topological and functional association of CGPs. **(a)**. Matrices of network distances between prostate CGPs (three categories: < 4 shortest distance shown in red; 4–6 shown in green; and > 6 shown in blue). CGP matrix clusters with significant enrichment in GO or KEGG annotations involved in tumorigenesis-related processes are indicated. **(b)**. Functional association of prostate CGPs. Cell communication (cluster C) and cell death (cluster G) biological processes are shown. Green, red and black nodes correspond to down-regulated, up-regulated, and non-differentially expressed proteins, respectively. Dashed circles and lines connect proteins common to both processes. Protein kinase C isozymes are denoted by the prefix PRKC.

Distances between CGPs can be represented in a matrix format where clusters are identified (Figure 4a and Additional file 7). We then investigated whether these topological associations or clusters of CGPs have functional implications for mechanisms of tumorigenesis. In this analysis, proportions of GO terms [18] and pathway (KEGG) [26] annotations were compared between clusters showing small network distances (≤ 3 shortest distance) and the remaining CGPs (≥ 4) in each matrix. Results showed that down-regulated CGPs in clusters participate in common biological processes or pathways involved in tumorigenesis (Table 3). Thus, the GO analysis revealed the coordinated down-regulation of CGPs involved in cell adhesion and cell communication processes, which would facilitate the metastatic behavior of cancer cells, and the coordinated down-regulation of CGPs involved in programmed cell death, which would in turn prolong cancer cell life and allow tumorigenesis to progress by accumulating genetic and molecular alterations. KEGG analysis revealed the coordinated down-regulation of pathways commonly associated with tumorigenesis, such as the extracellular matrix-receptor interaction pathway. It also revealed the coordinated down-regulation of pathways known to play a critical role in prostate carcinogenesis, for example the insulin signaling pathway [27].

**Table 3:** Topological and functional association of prostate CGPs

Non-redundant significant terms*	**GO level**	***P *value FDR-adjusted**
**Data set 1**		
**Cluster A**		
BP: Protein amino acid phosphorylation	8	9.12E-03
CC: Plasma membrane	4	2.25E-02
MF: Protein-tyrosine kinase activity	7	9.12E-03
		
**Cluster B**		
BP: Cell-matrix adhesion	5	4.29E-02
CC: Extracellular space	3	4.09E-04
MF: Metalloendopeptidase inhibitor activity	6	3.75E-03
		
**Cluster C**		
BP: Intracellular signaling cascade	5	3.74E-02
CC: Cytoskeleton	4	4.11E-02
MF: Protein-tyrosine kinase activity	7	1.76E-03
KEGG: T cell receptor signaling pathway		1.18E-02
Adherens junction		2.21E-02
Focal adhesion		4.11E-02
		
**Cluster D**		
BP: Macromolecule biosyntesis	5	3.17E-02
CC: Cytosolic ribosome	5	2.30E-02
MF: Structural constituent of ribosome	7	6.86E-03
KEGG: Ribosome		6.86E-03
		
**Data set 2**		
**Cluster E**		
MF: Purine nucleotide binding	4	1.51E-03
		
**Cluster F**		
CC: Extracellular space	3	8.00E-03
MF: Extracellular matrix structural constituent	3	8.00E-03
KEGG: Extracellular matrix receptor interaction		8.00E-03
		
**Cluster G**		
BP: Regulation of programmed cell death	5	4.32E-02
MF: Protein kinase activity	6	2.18E-02
KEGG: Insulin signaling pathway		4.62E-02
		
**Cluster H**		
BP: Phosphate transport	8	1.27E-02
CC: Extracellular space	3	1.10E-12
MF: Metalloendopeptidase inhibitor activity	6	3.05E-02
KEGG: Extracellular matrix receptor interaction		2.07E-03

*BP (Biological Process), CC (Cellular Component), MF (Molecular Function), KEGG (Kyoto Encyclopedia of Genes and Genomes)

Most up-regulated CGP topological associations did not show significant enrichment in GO or KEGG annotations, however, when all CGPs were considered together, both up- and down-regulated CGPs participating in common biological processes and pathways were found closely located in the network. For example, up- and down-regulated CGPs within and connecting cell communication and cell adhesion functions are protein kinase C isozymes (Figure 4b), which are well known regulators of cell proliferation and transformation of prostate epithelial cells [28]. This CGP organization might reflect a change in the flow of information between different processes so as to promote tumorigenesis.

## Discussion

A criterion of centrality for a particular node in a network can be given by local (degree) or by global (betweenness and closeness) measures. A higher degree does not necessarily mean that a node is more important for information exchange and propagation, so more global measures are needed than degree measures. The three measures of centrality therefore reflect the possibilities of a particular protein choosing alternative paths, acting as a broker between different proteins, for example connecting distinct complexes or signaling pathways, or being closer to any other proteins for information propagation. By virtue of centrality, the hundreds of differentially expressed proteins in tumors are likely to promote tumorigenesis at the interactome network level in a coordinated manner. Viewed alternatively, proteins with a less central position within the interactome network might not be able to have a global impact on the cellular behavior determined by the protein-protein interactions involved in cellular transformation.

Protein-protein interactions repositories are incomplete and not fully reliable, based on the observed selection and detection biases [20,21]. Gandhi et al. [12] demonstrated that there is minimal overlap across currently known experimental species interactome data sets. In addition, recent work by Mika and Rost [29] has shown that interactions are more conserved within species than across species and that homology transfers are only accurate at high levels of identity. These observations raise the question of the specificity and sensitivity of large-scale homology-based generated interactomes with respect to other approaches. The principle of conserved protein-protein interactions or "interologs" was first used by Matthews et al. [30] and subsequently extended by several authors [31-33]. The Jonsson et al. [11,22] data set used in the present study applied a new confidence score to predict interactions, which was based on both the level of homology and the amount of experimental data available that supported a particular interaction. By benchmarking the score the authors obtained relatively good percentages of sensitivity and specificity (~80–85%) for a reasonable cut-off [11], which indicates high reliability of the data set. This observation corroborates our results by replicating the findings with respect to centrality.

In addition to selection and detection biases, the limited coverage of current data sets relative to the anticipated complete human interactome suggests that results derived from any currently available set should be interpreted with an element of caution, as has been demonstrated for other well-established topology characteristics [34]. The results using the Jonsson et al. [22] data set show increased centrality for up-regulated CGPs and differential CC values that were not consistently observed when using other interaction sets. The larger size of this data set could reduce the effect of sampling and may facilitate the detection of weak effects. This apparent discrepancy will probably remain unexplained until larger coverage of the anticipated human experimental interactome has been obtained. Nevertheless, we analyzed hundreds of CGPs, most of which belonged to different sets across different cancer types, which makes this study less likely to present a bias in gene selection.

In a previous study focused on lung cancer, it was suggested that up-regulated CGPs in squamous lung tumors have higher connectivity [10], yet the same observation was not supported for down-regulated CGPs. This discrepancy could be due to the small number of samples profiled (five tumors and matched healthy tissues) but also to the examination of another interactome network generated mainly from computationally-generated interactions. On the other hand, the lung data set we used [17] has been extensively examined and validated, which suggest that the apparent centrality inconsistency of down-regulated CGPs is not due to the existence of different subsets of lung CGPs.

This study proposes a model for systems-level molecular mechanisms of tumorigenesis that includes the down-regulation of specific biological processes represented by topological associations of CGPs in the interactome network, combined with the up-regulation of particular proteins that could depend on the cell type, tumor type or tumor stage. Since we analyzed tumor panels, our results reflect average, possibly secondary molecular events in cancer. These changes are initiated by combinations of genetic alterations in tumor suppressor genes and oncogenes, which lead to extensive downstream variation of expression modules carrying specific functions in cancer cells [7,8]. The analysis of tumors ordered in stages would provide additional information on the systems-level molecular mechanisms of cancer progression. The final outcome of CGP organization could be a change in the flow of information, specific to each cancer type that will determine the neoplastic process. Centrality could then be used in combination with dynamic information (i.e., gene or pathway up- or down-regulation) to specifically disrupt cancer cell networks by disturbing proteins that are critical to both aspects.

## Conclusion

Taking into account the current limited coverage of the anticipated, complete human interactome, this study suggests that the proteins encoded by differentially expressed genes in tumors relative to healthy tissue counterparts occupy central positions in the interactome network. Our results suggest a systems-level tumorigenesis model that comprises the precise down-regulation of groups of topologically-associated proteins involved in particular functions, orchestrated with the up-regulation of specific proteins.

## Methods

### Human interactome network

In generating the human interactome network, a previously compiled data set was used, mainly containing experimentally demonstrated interactions compiled through a literature-curation process, combined with data from different types of experimental and computational evidence [12]. In our analyses, proteins with no assigned Entrez GeneID were excluded, thus yielding a final interactome network containing 7,388 proteins and 24,109 interactions. The network was analyzed using Cytoscape [35] and UCINET [36]. In removing network hubs, proteins were selected from the highest degree value (> 9 when analyzing prostate CGPs to > 19 when analyzing colorectal CGPs). The number of proteins/nodes removed from the network in each case was identical amongst hubs, CGPs and selected proteins with the same degree distribution as CGPs but with lower values of betweenness and closeness.

### Gene expression analysis

The GEPAS package [37] was used for the analysis of expression data. Background correction, normalization and averaging of expression values were performed with the Robust Multi-array Average (RMA) algorithm [38]. Differentially expressed genes between healthy and tumor samples were declared after the calculation of an empirical Bayes moderated t-statistic, and *P *values adjusted by false discovery rate of 5%. Previously analyzed colorectal data [16] were down-loaded from a public repository [19]. Data sets and probe lists are detailed in Table S1. The FatiScan tool [39] was used to assess enrichment of GO terms in the complete gene ranking according to the empirical Bayes moderated t-statistic.

### Topological analysis

The degree of a vertex or protein in the interactome network was calculated by counting the number of edge-ends at that node. Betweenness was calculated in accordance with Freeman's formulation [40]. Thus, betweenness centrality *C*_*B*_(*v*) for vertex *v *is calculated as follows:

CB(v)=∑s≠v≠t∈Vσst(v)σst
 MathType@MTEF@5@5@+=feaafiart1ev1aaatCvAUfKttLearuWrP9MDH5MBPbIqV92AaeXatLxBI9gBaebbnrfifHhDYfgasaacH8akY=wiFfYdH8Gipec8Eeeu0xXdbba9frFj0=OqFfea0dXdd9vqai=hGuQ8kuc9pgc9s8qqaq=dirpe0xb9q8qiLsFr0=vr0=vr0dc8meaabaqaciaacaGaaeqabaqabeGadaaakeaacqWGdbWqdaWgaaWcbaGaemOqaieabeaakiabcIcaOiabdAha2jabcMcaPiabg2da9maaqababaWaaSaaaeaaiiGacqWFdpWCdaWgaaWcbaGaem4CamNaemiDaqhabeaakiabcIcaOiabdAha2jabcMcaPaqaaiab=n8aZnaaBaaaleaacqWGZbWCcqWG0baDaeqaaaaaaeaacqWGZbWCcqGHGjsUcqWG2bGDcqGHGjsUcqWG0baDcqGHiiIZcqWGwbGvaeqaniabggHiLdaaaa@4C80@

where *σ*_*st *_is the number of shortest geodesic paths from *s *to *t *and *σ*_*st *_(*v*) the number of shortest geodesic paths from *s *to *t *that pass through the vertex *v*. This value was normalized by dividing by (*n *-1) × (*n *- 2), where *n *is the number of vertices. Closeness centrality was calculated according to Sabidussi's formulation [41]. Thus, the closeness *C*_*C*_(*v*) for a vertex *v *is the reciprocal of the sum of geodesic distances to all other vertices in graph *G*, and is calculated as follows:

CC(v)=1∑t∈VdG(v,t)
 MathType@MTEF@5@5@+=feaafiart1ev1aaatCvAUfKttLearuWrP9MDH5MBPbIqV92AaeXatLxBI9gBaebbnrfifHhDYfgasaacH8akY=wiFfYdH8Gipec8Eeeu0xXdbba9frFj0=OqFfea0dXdd9vqai=hGuQ8kuc9pgc9s8qqaq=dirpe0xb9q8qiLsFr0=vr0=vr0dc8meaabaqaciaacaGaaeqabaqabeGadaaakeaacqWGdbWqdaWgaaWcbaGaem4qameabeaakiabcIcaOiabdAha2jabcMcaPiabg2da9maalaaabaGaeGymaedabaWaaabeaeaacqWGKbazdaWgaaWcbaGaem4raCeabeaakiabcIcaOiabdAha2jabcYcaSiabdsha0jabcMcaPaWcbaGaemiDaqNaeyicI4SaemOvayfabeqdcqGHris5aaaaaaa@4250@

Hierarchy and constraint were calculated using Burt's formulation [42]. Constraint is a summary measure that indicates the level of independence of a node from its neighbourhood, depending on the number of edges that connect it to neighbour nodes. Thus, constraint is calculated as follows:

cij=(pij+∑qpiqpqj)2
 MathType@MTEF@5@5@+=feaafiart1ev1aaatCvAUfKttLearuWrP9MDH5MBPbIqV92AaeXatLxBI9gBaebbnrfifHhDYfgasaacH8akY=wiFfYdH8Gipec8Eeeu0xXdbba9frFj0=OqFfea0dXdd9vqai=hGuQ8kuc9pgc9s8qqaq=dirpe0xb9q8qiLsFr0=vr0=vr0dc8meaabaqaciaacaGaaeqabaqabeGadaaakeaacqWGJbWydaWgaaWcbaGaemyAaKMaemOAaOgabeaakiabg2da9maabmaabaGaemiCaa3aaSbaaSqaaiabdMgaPjabdQgaQbqabaGccqGHRaWkdaaeqaqaaiabdchaWnaaBaaaleaacqWGPbqAcqWGXbqCaeqaaOGaemiCaa3aaSbaaSqaaiabdghaXjabdQgaQbqabaaabaGaemyCaehabeqdcqGHris5aaGccaGLOaGaayzkaaWaaWbaaSqabeaacqaIYaGmaaaaaa@45DF@

for *q *≠ *i, j*, where *p*_*ij *_is the proportion of node *i *connections to *j*. Hierarchy is the extent to which constraint is concentrated in a single node and is calculated as follows:

H=∑j(cijC/N)ln⁡(cijC/N)Nln⁡N
 MathType@MTEF@5@5@+=feaafiart1ev1aaatCvAUfKttLearuWrP9MDH5MBPbIqV92AaeXatLxBI9gBaebbnrfifHhDYfgasaacH8akY=wiFfYdH8Gipec8Eeeu0xXdbba9frFj0=OqFfea0dXdd9vqai=hGuQ8kuc9pgc9s8qqaq=dirpe0xb9q8qiLsFr0=vr0=vr0dc8meaabaqaciaacaGaaeqabaqabeGadaaakeaacqWGibascqGH9aqpdaWcaaqaamaaqababaWaaeWaaeaadaWcaaqaaiabdogaJnaaBaaaleaacqWGPbqAcqWGQbGAaeqaaaGcbaGaem4qamKaei4la8IaemOta4eaaaGaayjkaiaawMcaaiGbcYgaSjabc6gaUnaabmaabaWaaSaaaeaacqWGJbWydaWgaaWcbaGaemyAaKMaemOAaOgabeaaaOqaaiabdoeadjabc+caViabd6eaobaaaiaawIcacaGLPaaaaSqaaiabdQgaQbqab0GaeyyeIuoaaOqaaiabd6eaojGbcYgaSjabc6gaUjabd6eaobaaaaa@4BDB@

The cluster coefficient is the local density of a node's connections and is defined as the ratio between the observed number of connections *Li *and the total number of possible connections for a particular node *i*, *ki *(*ki *- 1). Thus, the clustering coefficient is calculated as follows:

C(i)=2Liki(ki−1)
 MathType@MTEF@5@5@+=feaafiart1ev1aaatCvAUfKttLearuWrP9MDH5MBPbIqV92AaeXatLxBI9gBaebbnrfifHhDYfgasaacH8akY=wiFfYdH8Gipec8Eeeu0xXdbba9frFj0=OqFfea0dXdd9vqai=hGuQ8kuc9pgc9s8qqaq=dirpe0xb9q8qiLsFr0=vr0=vr0dc8meaabaqaciaacaGaaeqabaqabeGadaaakeaacqWGdbWqcqGGOaakcqWGPbqAcqGGPaqkcqGH9aqpdaWcaaqaaiabikdaYiabdYeamnaaBaaaleaacqWGPbqAaeqaaaGcbaGaem4AaSMaemyAaKMaeiikaGIaem4AaSMaemyAaKMaeyOeI0IaeGymaeJaeiykaKcaaaaa@3E85@

The Bron and Kerbosch algorithm [43] was used to find all cliques greater than a specified size [44].

To assess significance, the non-parametric Mann-Whitney *U *test was used to determine whether the median of the centrality measures was different between protein sets. To assess significance, the properties of CGPs were also compared to equivalent, randomly selected protein sets in the interactome. One thousand random iterations were performed in each case. Subsequently, average values, confidence intervals and empirical *P *values were obtained.

### Functional association analysis

The Stats and Graphics packages in R [45] were used to analyze and plot the matrix of network distances between CGPs, respectively. Hierarchical clustering with an average linkage method was applied to the matrix so as to arrange CGPs according to their network distances. The FatiGO+ tool [46] was then used to assess GO and KEGG annotations enrichment between the set of CGPs within a particular cluster and the remaining CGPs in the same matrix. Calculated *P *values were adjusted by FDR, taking into account the total number of genes interrogated in each case.

## Authors' contributions

PH participated in the study design, compiled and analyzed the gene expression and protein-protein interaction data sets, and helped to draft the manuscript. JHC participated in the study design and performed microarray analyses. DM, FAS and JV helped with microarray and statistical analyses. LG and GC participated in scientific discussions. GC provided institutional and grant supports. MAP and JD conceived the study. JHC and JD helped to draft the manuscript. MAP designed and coordinated the study, and wrote the original and final versions of the manuscript. All authors read and approved the final version of the manuscript.

## Supplementary Material

Additional File 1 **(Figure S1)**. Human interactome network characteristics. Scale-free and degree distribution. The probability that a protein is connected to *k *other proteins is described by *P(k)*. Click here for file

Additional File 2 **(Table S1)**. Gene expression data set descriptions and differentially expressed probe sets. Click here for file

Additional File 3 **File 3 (Figure S2)**. FatiScan analysis of prostate gene expression data sets. Annotations of Biological Process, Cellular Component and Molecular Function GO terms (level 3) in the complete gene ranking are shown. Click here for file

Additional File 4 **File 4 (Table S2)**. Statistical analysis results for centrality, constraint, and cluster coefficient using the interactome data set described by Gandhi et al. [12]. Click here for file

Additional File 5 **File 5 (Figure S3)**. Centrality of down- (green arrows) and up-regulated (red arrows) prostate CGPs. Results of the Mann-Whitney *U *test (MW) are shown at the top right in each box. Results of comparing each centrality measure between prostate CGPs (vertical arrow; mean value) and 1,000 equivalent randomly selected protein sets (curves; mean values) (data sets 1 [14] and 2 [15]) are also shown. CGPs mean values and 95% confidence intervals (CI), as well consequent empirical *P *values are shown. Click here for file

Additional File 6 **File 6 (Table S3)**. Statistical analysis results for centrality, constraint, and cluster coefficient using three interactome data sets: i/ *in vivo *experimental interactions only; ii/ interactions with two or more *in vivo *or *in vitro *experimental evidences as compiled by Gandhi et al. [12]; and iii/ computationally-generated interactions using a homology-based method [22]. Click here for file

Additional File 7 **File 7 (Figure S4)**. Topological and functional association of lung and colorectal CGPs. Matrices of distances between CGPs (three categories: < 4 shown in red; 4–6 shown in green; and > 6 shown in blue) and GO and KEGG annotations enriched in matrix clusters are shown. Click here for file
