# Supplementary figures and images for: Evidence for systems-level molecular mechanisms of tumorigenesis
Source: BMC Genomics. 2007 Jun 20;8:185. doi: 10.1186/1471-2164-8-185 (PMC1929080; doi:10.1186/1471-2164-8-185)

### Scale-free

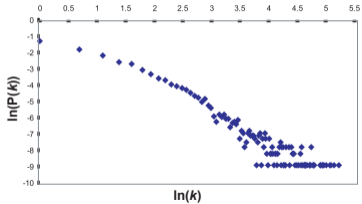

### Degree distribution

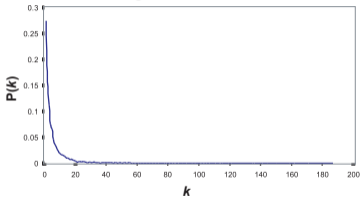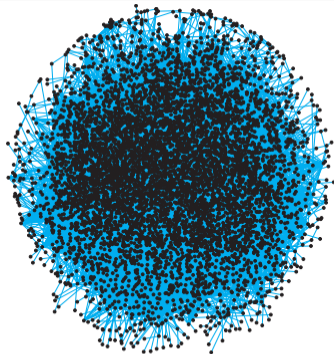

**Human interactome network**

**7,388 Proteins**

**24,109 Interactions**

Supplement: Additional File 1 — (Figure S1). Human interactome network characteristics. Scale-free and degree distribution. The probability that a protein is connected to k other proteins is described by P(k). [file 1471-2164-8-185-S1.pdf]

FatiScan Results  
Prostate data set 1

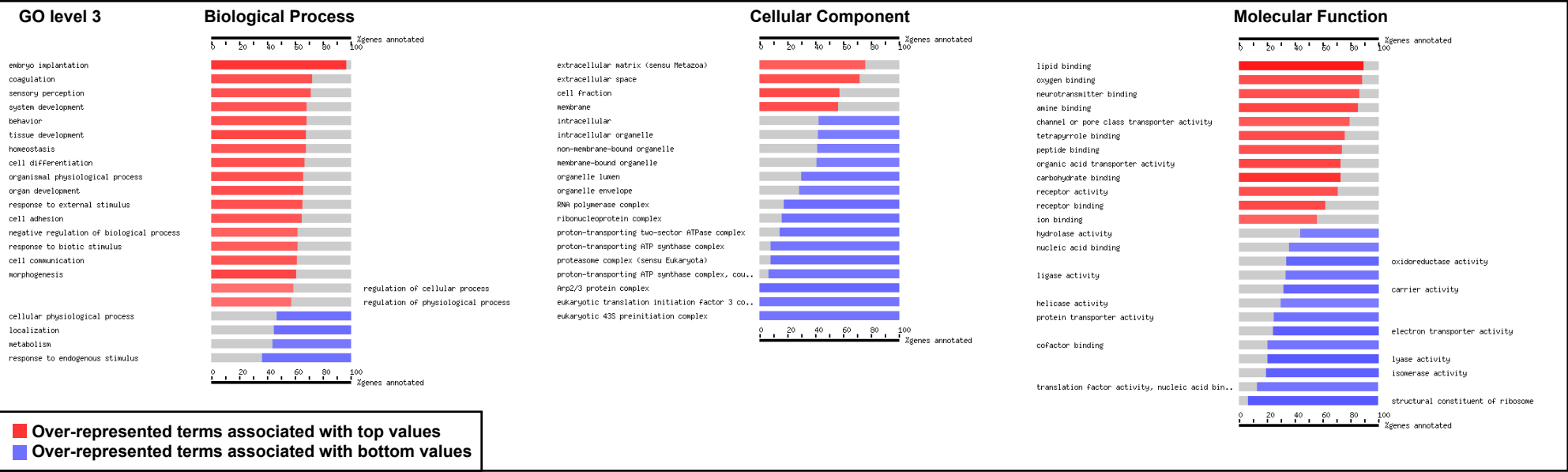

Supplement: Additional File 3 — File 3 (Figure S2). FatiScan analysis of prostate gene expression data sets. Annotations of Biological Process, Cellular Component and Molecular Function GO terms (level 3) in the complete gene ranking are shown. [file 1471-2164-8-185-S3.pdf]

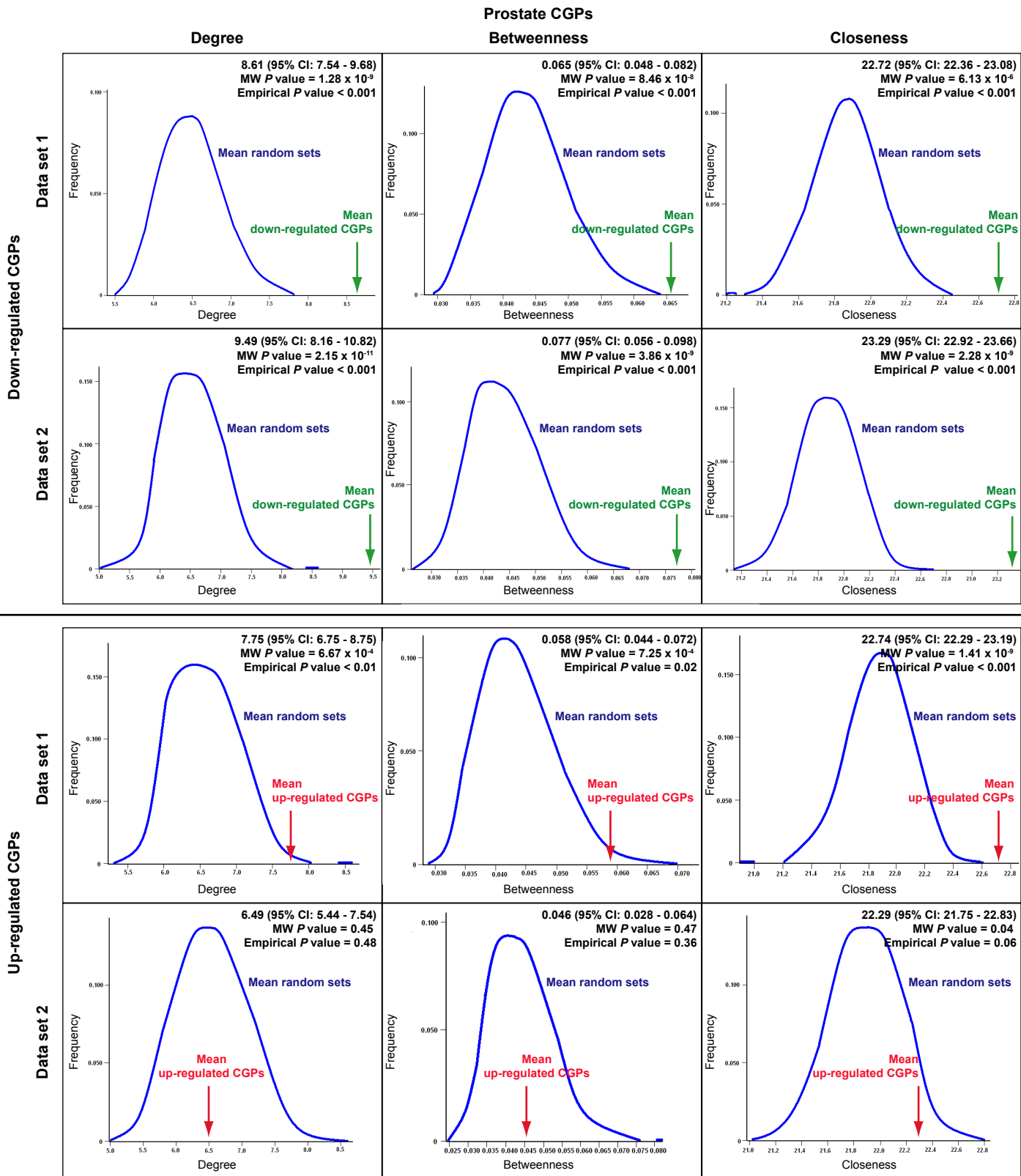

Supplement: Additional File 5 — File 5 (Figure S3). Centrality of down- (green arrows) and up-regulated (red arrows) prostate CGPs. Results of the Mann-Whitney U test (MW) are shown at the top right in each box. Results of comparing each centrality measure between prostate CGPs (vertical arrow; mean value) and 1,000 equivalent randomly selected protein sets (curves; mean values) (data sets 1 [14] and 2 [15]) are also shown. CGPs mean values and 95% confidence intervals (CI), as well consequent empirical P values are shown. [file 1471-2164-8-185-S5.pdf]
