# Supplementary material for: Evidence for systems-level molecular mechanisms of tumorigenesis
Source: BMC Genomics. 2007 Jun 20;8:185. doi: 10.1186/1471-2164-8-185 (PMC1929080; doi:10.1186/1471-2164-8-185)
Supplement: Additional File 7 — File 7 (Figure S4). Topological and functional association of lung and colorectal CGPs. Matrices of distances between CGPs (three categories: < 4 shown in red; 4–6 shown in green; and > 6 shown in blue) and GO and KEGG annotations enriched in matrix clusters are shown. [file 1471-2164-8-185-S7.pdf]

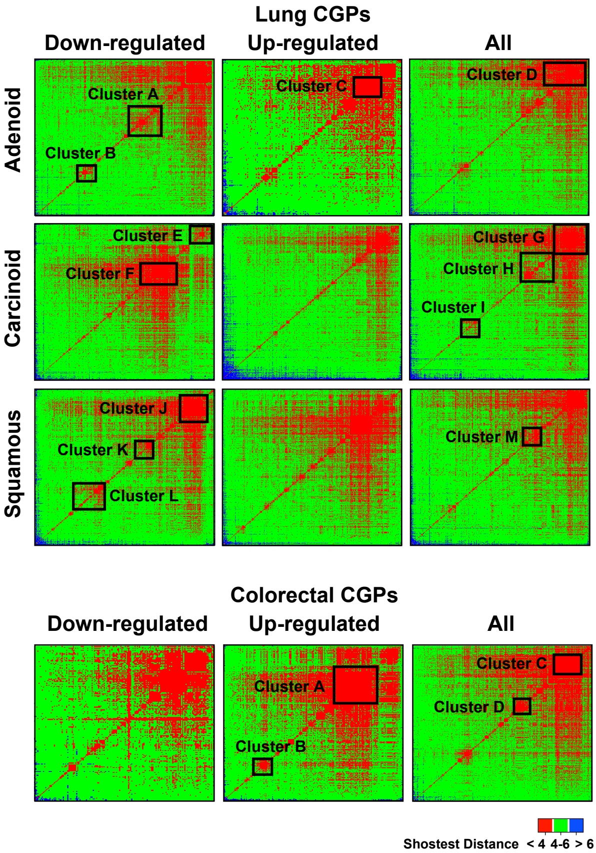

Topological and functional association of lung CGPs

Non-redundant significant terms*	GO level	*P* value FDR-adjusted
**Cluster A**		
BP: Regulation of transcription, DNA-dependent	8	4.17E-02
CC: Nucleus	4	1.04E-02
MF: Transcription factor activity	3	1.44E-02
**Cluster B**		
CC: Extracellular space	3	1.09E-02
MF: Extracellular matrix structural constituent	3	4.62E-02
KEGG: Complement and coagulation cascades		4.15E-02
**Cluster C**		
CC: Collagen	4	4.40E-02
**Cluster D**		
BP: Organismal physiological process	3	4.93E-02
CC: Extracellular space	3	2.93E-02
MF: Metalloendopeptidase inhibitor activity	6	3.05E-02
KEGG: Complement and coagulation cascades		4.32E-02
**Cluster E**		
BP: Intracellular signaling cascade	5	4.84E-03
MF: Protein kinase activity	6	4.14E-02
**Cluster F**		
BP: Response to stress	3	1.52E-02
CC: Extracellular space	3	4.07E-02
MF: Protease inhibitor activity	4	4.90E-02
KEGG: Extracellular matrix receptor interaction		3.09E-03
**Cluster G**		
BP: Signal transduction	4	4.34E-02
MF: Purine nucleotide binding	4	8.00E-03
KEGG: T cell receptor signaling pathway		4.34E-02
**Cluster H**		
BP: Phosphate metabolism	6	4.65E-02
**Cluster I**		
BP: Cell adhesion	3	4.67E-03
CC: Extracellular space	3	3.78E-05
MF: Metalloendopeptidase inhibitor activity	6	1.11E-02
KEGG: Complement and coagulation cascades		1.11E-02
Extracellular matrix receptor interaction		3.31E-03
**Cluster J**		
BP: Intracellular signaling cascade	5	1.53E-02
**Cluster K**		
BP: Regulation of transcription, DNA-dependent	8	6.00E-03
CC: Nucleus	4	8.99E-05
MF: Transcription factor activity	3	3.14E-04
**Cluster L**		
BP: Response to stress	3	2.65E-02
CC: Extracellular component	3	2.65E-02
MF: Enzyme inhibitor activity	3	4.16E-02
**Cluster M**		
BP: Regulation of transcription, DNA-dependent	8	2.97E-02
CC: Nucleus	4	7.17E-05
MF: Transcription factor activity	3	2.97E-02

*BP (Biological Process), CC (Cellular Component), MF (Molecular Function), KEGG (Kyoto Encyclopedia of Genes and Genomes pathway annotation)

Topological and functional association of colorectal CGPs

Non-redundant significant terms*	GO level	*P* value FDR-adjusted
**Cluster A**		
CC: Extracellular matrix	3	1.13E-04
**Cluster B**		
MF: Structural component of ribosome	3	1.53E-02
KEGG: Ribosome		2.60E-02
**Cluster C**		
CC: Extracellular matrix	3	1.99E-03
**Cluster D**		
MF: Structural component of ribosome	3	1.17E-02

*BP (Biological Process), CC (Cellular Component), MF (Molecular Function), KEGG (Kyoto Encyclopedia of Genes and Genomes pathway annotation)
